# An association study on contrasting cystic fibrosis endophenotypes recognizes *KRT8 *but not *KRT18 *as a modifier of cystic fibrosis disease severity and CFTR mediated residual chloride secretion

**DOI:** 10.1186/1471-2350-12-62

**Published:** 2011-05-06

**Authors:** Frauke Stanke, Silke Hedtfeld, Tim Becker, Burkhard Tümmler

**Affiliations:** 1 Department of Pediatrics, Hannover Medical School, Hannover, Germany; 2 Institute of Medical Biometry, Informatics and Epidemiology, University of Bonn, Bonn, Germany

## Abstract

**Background:**

F508del-CFTR, the most frequent disease-causing mutation among Caucasian cystic fibrosis (CF) patients, has been characterised as a mutant defective in protein folding, processing and trafficking. We have investigated the two neighbouring cytokeratin genes *KRT8 *and *KRT18 *in a candidate gene approach to ask whether variants in *KRT8 *and/or *KRT18 *modify the impaired ion conductance known as the CF basic defect, and whether they are associated with correct trafficking of mutant CFTR and disease severity of CF.

**Methods:**

We have selected contrasting F508del-*CFTR *homozygous patient subpopulations stratified for disease severity, comparing 13 concordant mildly affected sib pairs vs. 12 concordant severely affected sib pairs, or manifestation of the CF basic defect in intestinal epithelium, comparing 22 individuals who exhibit CFTR-mediated residual chloride secretion vs. 14 individuals who do not express any chloride secretion, for an association. The *KRT8*/*KRT18 *locus was initially interrogated with one informative microsatellite marker. Subsequently, a low density SNP map with four SNPs in KRT8 and two SNPs in KRT18, each selected for high polymorphism content, was used to localize the association signal.

**Results:**

*KRT8*, but not *KRT18*, showed an association with CF disease severity (P_best _= 0.00131; P_corr _= 0.0185) and CFTR mediated residual chloride secretion (P_best _= 0.0004; P_corr _= 0.0069). Two major four-marker-haplotypes spanning 13 kb including the entire *KRT8 *gene accounted for 90% of chromosomes, demonstrating strong linkage disequilibrium at that locus. Absence of chloride secretion was associated with the recessive haplotype 1122 at rs1907671, rs4300473, rs2035878 and rs2035875. The contrasting haplotype 2211 was dominant for the presence of CFTR mediated residual chloride secretion. In consistency, the *KRT8 *haplotype 2211 was associated with mild CF disease while 1122 was observed as risk haplotype. Analysis of microsatellite allele distributions on the SNP background suggests that the mild *KRT8 *haplotype 2211 is phylogenetically older than its severe counterpart.

**Conclusions:**

The two opposing *KRT8 *alleles which have been identified as a benign and as a risk allele in this work are likely effective in the context of epithelial cell differentiation. As the mild *KRT8 *allele is associated with CFTR mediated residual chloride secretion among F508del-*CFTR *homozygotes, the KRT8/KRT18 heterodimeric intermediary filaments of the cytoskeleton apparently are an essential component for the proper targeting of CFTR to the apical membrane in epithelial cells.

## Background

Cystic fibrosis (CF, OMIM#219700) is an autosomal recessive monogenic disease, caused by two defective copies of the cystic fibrosis transmembrane conductance regulator (*CFTR*) gene [1]. *CFTR*, encoding a chloride- and bicarbonate transporter, is expressed in epithelial cells [1]. The impaired ion conductance of CFTR expressing epithelia, known as the CF basic defect, can be addressed for analytical and diagnostic purposes in the sweat gland [1], intestinal [2] and respiratory [3] tissue using in-vivo and ex-vivo methods. CFTR dysfunction leads to a generalized exocrinopathy whereby symptoms of the gastrointestinal and the respiratory tracts dominate the clinical manifestation in CF [1].

CF is characterized by allelic heterogeneity, but among patients of Caucasian descent, 70-80% of disease-causing alleles are F508del-*CFTR*. Hence, about half of the patient population is F508del-*CFTR *homozygous [1]. In spite of the homogeneity of these patients with respect to the major disease-causing gene, the course of disease varies considerably due to environmental factors and genes other than *CFTR *[4,5]. These so-called modifying genes are studied by several researchers [6] using candidate-gene based [5,7] and genome-wide approaches [8,9].

F508del-CFTR is known as a mutant defective in protein folding, processing and trafficking [10]. While the CF basic defect in the sweat gland shows consistently pathologically elevated sweat chloride concentrations for all CF patients homozygous for F508del-*CFTR *[1], the manifestation of the basic defect in the respiratory and gastrointestinal tract is variable in these patients, reflecting the diverse clinical course [11,12]. In excised rectal suction biopsies analysed by intestinal current measurement [2], the CFTR mediated chloride current ranges from not detectable to subnormal residual chloride secretion [11,12].

As *CFTR *is expressed epithelial cells of many tissues throughout the body, CF is a complex multi-organ disease [1] whereby the severity of some pathological manifestations are correlated. Patients who are pancreatic sufficient are less susceptible to chronic colonization of the airways by opportunistic pathogens such as *Pseudomonas aeruginosa *than pancreatic insufficient patients [13,14]. F508del-*CFTR *homozygotes who display CFTR mediated residual chloride secretion in the airways or the intestine have a milder clinical phenotype in comparison to F508del-*CFTR *homozygotes who do not express any chloride secretion in their tissues [12]. These two examples and other similar observations suggest that the variability of the individual disease manifestations are based on overlapping or shared causes. The corresponding concept from the field of epidemiology describes such manifestations as endophenotypes.

The term endophenotypes was coined by the entomologists John and Lewis who derived the concept in the 1960s studying the geographical distributions of grasshopper populations [15]. At the present time, endophenotypes are taken into account by genetic epidemiologists who analyze inherited factors that determine psychiatric diseases such as schizophrenia, depression or bipolar disorder [16]. Using endophenotypes is considered advantageous to the identification of causative genes because these are likely to be determined be fewer genes and less likely prone to environmental perturbations than the global disease manifestation [16]. Hence, as the CF basic defect is a manifestation of CFTR dysfunction at the cellular level and likely to be less complex than a global disease phenotype such as lung function, we hypothesize that measures of impaired CFTR function can be used to get insight into CF modifying genes.

We hypothesized that the cellular protein network that governs CFTR trafficking and processing [17,18], including the cytokeratin I/cytokeratin II pair KRT18/KRT8 which heteropolymerize to form intermediate-sized filaments in the cytoplasm of epithelial cells [19], might determine the diversity in basic defect manifestations among F508del-*CFTR *homozygous individuals. We have recently described our candidate gene based analysis for 52 target genes on 16 different chromosomes using a total of 182 genetic markers [5]. Here, we report on unpublished findings on the cytokeratin gene cluster on 12q13 among European CF twins and siblings with contrasting clinical and basic defect endophenotypes genotyped at one informative intergenic microsatellite marker and six single nucleotide polymorphisms intragenic to the two neighbouring genes *KRT8 *and *KRT18*.

## Methods

### Study population

We have carried out an association study comparing F508del-CFTR homozygous cystic fibrosis patient subsets selected for an extreme clinical phenotype and/or their manifestation of impaired ion conductance. The study population and the selection criteria for cases and references of the association study has been described in detail elsewhere [5]. Briefly, genotyping data from 101 CF families, 85 of which are a subgroup of the twin and sibling study panel of 466 twin and sibling pairs, was used for the association study [5]. 16 F508del-CFTR homozygous singletons with known basic defect and their parents were included into the analysis of the manifestation of the CF basic defect. These 16 patients were recruited from the local CF clinic at Hannover Medical School for a study on the manifestation of the basic defect in excised intestinal biopsies and subsequent CFTR protein analysis and chip-based transcriptome analysis [5,20].

All patients have been enrolled into the association study based on their extreme clinical and/or their basic defect phenotype as characterized by intestinal current measurement or nasal potential difference measurement of the impaired ion conductance in CF of the intestinal and respiratory epithelium, respectively [5]. While the entire data set of two indels, 101 SNPs and 79 microsatellites, which include the KRT8/KT18 locus as a candidate gene, has recently been published [5], this work gives details on *KRT8 *and *KRT18 *that have not been reported elsewhere. In particular, we now report on the results of our fine-mapping approach using a low density SNP map, assess the mode of inheritance and name the risk and the benign allele at the KRT8/KRT18 locus. These results have not been detailed in our previous work.

### Ethics approval

This study consists of the three subprojects no. 2771, 3739 and 4656 each of which was approved by the ethics committee of the Medizinische Hochschule Hannover prior to the start of the respective subproject. Continuation of the study for the time period from July 1, 2009 until June 30, 2013 was granted by the ethics committee by March 03, 2009. Patients who have been enrolled into the association study have all given informed consent that their DNA is analyzed for genetic modifiers of CF disease in their DNA.

### Case-reference populations to investigate association to CF disease severity

Twin and sibling pairs with cystic fibrosis were recruited from 158 CF clinics in 14 European countries [5]. Basic clinical data such as actual weight, height and forced expiratory volume in one second was inquired from the treating physician using a one-page questionnaire [5]. To describe the severity of the disease with one parameter only which accounts for the severity of CF disease in the two major afflicted organ systems, i.e. the gastrointestinal and pulmonary tract, these basic clinical parameters were combined in a composite parameter using a ranking algorithm [21]. For this purpose, weight and height were converted to weight as % of predicted weight for height (wfh%) and FEV1 values were converted to CF population centiles for FEV1 as % of predicted value (FEVPerc) [21]. Centiles of wfh% and FEVPerc were age independent in the analyzed CF patient population [21]. Next, wfh% and all FEVPerc values for the entire study population were converted to rank numbers [21]. We have chosen this approach in order to describe overall CF disease severity assuming an equal weight of the anthropometric and the pulmonary component.

In order to validate that the composite parameter is suitable to detect the influence of inherited factors on CF disease, dizygous and monozygous twin pairs were compared in their intrapair differences in FEVPerc, wfh% and the composite parameter derived thereof. Intrapair discordance was significantly lower in monozygous twin pairs as long as the composite parameter was considered, while no association of intrapair concordance and twin zygosity was seen when only wfh% or only FEVPerc were examined [21]. Interpreting the higher concordance of monozygous twin pairs as an indication of inherited factors that determine the phenotype, we concluded from this finding that the composite parameter was more sensitive to detect inherited factors than either of the individual clinical parameters wfh% and FEVPerc. Consequently, we relied on the composite parameter to select patient pairs with extreme clinical phenotypes for the association study.

Based on the clinical disease severity of the individual siblings and the intrapair discordance, concordant mildly affected pairs (CON+; two sibs with similar and mild phenotype) and concordant severely affected pairs (CON-; two sibs with similar and severe phenotype) were defined [21]. As described previously [22], severely affected sibs and mildly affected sibs differ significantly in both clinical parameters utilized by the ranking algorithm. Respectively, mean [inner quartiles; range] were as follows: for the intrapair sum of wfh% -- 177[170-187;142-191] for concordant severely affected sib pairs and 212[206-217;202-229] for concordant mildly affected patient pairs; p < 0.001 (comparison carried out by Mann-Whitney rank test); for the intrapair sum of FEVPerc -- 43[18-66;9-97] for concordant severely affected sib pairs and 116[85-143;60-190] for concordant mildly affected patient pairs; p < 0.001 (comparison carried out by Mann-Whitney rank test) [22]. *KRT8 *and *KRT18 *markers were interrogated for their association with disease severity using the phenotypic contrast between concordant/mildly (CON+) affected patient pairs (26 patients, 13 affected patient pairs) and concordant/severely (CON-) affected patient pairs (23 patients, 12 affected patient pairs, one thereof represented by one sibling only) [5].

### Case-reference populations to investigate association to the manifestation of the CF basic defect

We have used intestinal current measurements (ICM) to characterize the patients' CF basic defect, defined as impaired or absent CFTR-mediated chloride secretion in CFTR-expressing tissues [1], in excised rectal suction biopsies [2]. ICM measurements on twins and sibs have been undertaken between 1997 and 1999 at selected CF core centers in Hannover, Innsbruck, London, Rotterdam, and Verona [11,12]. In addition, 16 F508del-*CFTR *homozygous singletons with known basic defect were recruited from the local CF center at the Hannover Medical School and included in the study [5]. Secretagogues such as carbachol, 8-bromo-adenosine-3',5'-cyclic monophosphate (cAMP) and histamine that activate or block ion channels, ion exchangers or components of the cellular signal transduction pathways were applied to excised rectal suction biopsies mounted in a micro-Ussing chamber to discriminate between patients with and without residual chloride secretion [2]. As ICM is an ex-vivo method applied to patient's biopsies, the toxic compound DIDS (4,48-diisothiocyanostilbene-2,28-disulfonic acid) which has been reported to block chloride channels other than CFTR, could be used to differentiate between CFTR-mediated residual chloride secretion and chloride secretion through alternative channels [2,5].

When analysed by ICM, non-CF controls display upon stimulation by carbachol or histamine short circuit currents (Isc) that exclusively monitor the Cl^- ^secretory response mediated by CFTR, because the obligatory K^+ ^efflux response response is not visible in the presence of fully functional CFTR [2]. For the diagnosis of CF, both the direction and the magnitude of Isc is used. Typically, CF patients display a biphasic response to carbachol and histamine as the fast K^+ ^current and the slower Cl^- ^current, both visible in classical CF cases, show opposite directions [2]. Responses range from 0% Cl^- ^response (equivalent to 100% K^+ ^signal) to 100% Cl^- ^response (equivalent to 0% K^+ ^signal) among F508del-CFTR homozygotes [11]. In the absence of a K^+ ^signal, a Cl^- ^secretory response below 10 μA/cm^2 ^is taken as an indicator of CFTR dysfunction and is consistent with CF [2]. The judgment of whether or not a recording shows evidence of residual CFTR activity is based on the relative proportions of the K^+ ^and the Cl^- ^signals in the biphasic response to carbachol and to histamine after the application of DIDS [11]. No evidence of a chloride secretory current values in both cases is interpreted as absence of CFTR function. Residual Cl^- ^secretion that is mediated by CFTR is assumed if both, carbachol and histamine response after the incubation with DIDS show a proportion of at least 40% of Cl^- ^secretory response (equivalent to 60% or less of K+ response) in the presence of a cAMP-mediated Cl^-^secretory response [11].

The cystic fibrosis basic defect assessed by intestinal current measurement was evaluated by comparing a set of 14 patients devoid of residual chloride secretion as controls to 22 patients who exhibit CFTR-mediated residual chloride secretion. These patients were either unrelated F508del-*CFTR *homozygotes or enrolled from a sib pair by an index-case strategy so that cases and controls for this comparison are unrelated [5].

### Data evaluation

Genetic data for the association study was evaluated using the FAMHAP software package [23] which allows family-based analysis [24,25] and accepts data evaluation in association studies on unrelated individuals as well as on affected sib pairs [23]. Case and reference population, as specified above, were analysed for an association of KRT8/KRT18 markers with the manifestation of CF disease severity and the manifestation of impaired ion conductance. Genotyping data was evaluated by comparing cases and references with respect to allele and haplotype as well as genotype and diplotype distributions.

All case-reference comparisons were carried out using 10,000 Monte-Carlo simulated data sets [23-25]. The analysis of more than one marker per locus is corrected for multiple testing by haplotype permutation [25]. For this purpose, the entire data set of cases and references is used to estimate haplotype frequencies [23]. Haplotype, or, in cases of non-informative phase or haplotype uncertainty, weighted haplotype explanation lists are assigned to each individual whereby the haplotype frequencies of the entire data set are taken into account to compute the conditional likelihood weights [23]. Permutation is done by randomly assigning the affection status to the individuals in each replication whereby the ratio of cases and controls is kept constant [23]. For the comparison of case sib pairs to reference sib pairs, the affection status is permuted or not with equal chance for both siblings simultaneously [23-25].

P-values for comparison of n-marker-haplotype and all marker subsets derived thereof are computed as s/n, where n is the number of permutation replicates, and where s is the number of permutation replicates leading to a test statistic higher than or equal to that of the real data [23]. Similarly, diplotype distributions between cases and controls are compared whereby a diplotype is a haplotype pair of the individual [23-25]. Reported P values are: P_raw_, referring to a computed P value of a single marker or a marker subset, P_best_, referring to the best observed P_raw _value, and P_corr_, referring to the P value of the entire marker set that is corrected for multiple testing. The adjustment for multiple testing properly accounts for LD within the Monte-Carlo simulation framework that evaluates the corrected signficance minP, the smallest observed raw P-value [24]. The computational details of the minP principle have been described elsewhere [24].

To allow a comparable assignment of weighted haplotype explanations in all subpopulations, the entire genotyping data of 101 CF families were provided as training set to FAMHAP for all case-reference comparisons [5].

### Genotyping

*KRT8 *and *KRT18 *markers have been genotyped as described before [5]. For microsatellite marker development, the genomic sequence was retrieved from the NCBI database selecting 200.000 bp upstream and downstream of *KRT8*. The primary sequence was analyzed with the program TandemRepeatFinder to reveal repetitive sequences. After in-silico analysis, priority was given to a sequence with high numbers of motif copies in the reference sequence because this marker is more likely to be polymorphic than short repeats, and to a sequence which is located between *KRT8 *and *KRT18*. The selected dinucleotide repeat KRT8Sat with the reference sequence (CA)_12_(TA)(CA)_2_(TA)(CA)_4 _starting at position 15442813 on contig NT_029419 was typed by polymerase-chain reaction amplification using unlabeled primer 5'-AAATGAGTGAATAAACATCACACG and biotin-labelled primer 5'-CCTTCAGATGTAGAGGGACGA [5]. PCR products were visualized by direct blotting electrophoresis on a high-resolution polyacrylamide gel and chemoluminescence detection of biotinylated PCR products as described elsewhere [5]. Application of the PCR amplicon for routine genotyping purposes was judged based on unrelated control samples. Alleles were scored by size in arbitrary repeat units whereby an invariant set of controls was used for calibration on all gels.

SNPs typed by PCR-RFLP were selected from internet resources whereby priority was given to markers based on their position on the physical map, aiming for intermarker intervals at *KRT8 *and *KRT18 *of more than 2000 bp and less than 10.000 bp, and the allele frequencies published for the European-Caucasian population, aiming for maximal polymorphism information content through selecting markers with equal frequencies of both alleles. SNPs were typed by polymerase-chain reaction with subsequent restriction digest (PCR-RFLP) using an enzyme that discriminates between the two alleles because the SNP alters the enzyme's recognition site. Application of the PCR-RFLP primer and enzyme combination for routine genotyping and informativity of the SNP was verified by genotyping unrelated control samples. Primers and restriction enzymes were as follows: rs1907671: 5'-GTTCTGCTCACCCCTTCCTC and 5'-AACTCTTTCCTTTTGGGGAGA with HaeIII; rs4300473: 5'-TGATCTGGGCTAAGGTGGTC and 5'-GGTGCTTCCTCTTCCTTTCC with AvaII; rs2035878: 5'-GAGATCAACTTCCTCCAGCAG and 5'-GCCTCTGGTTGAGTCTCAGG with AlwNI; rs2035875: 5'-TGAATTAAGAGAAAAGACGAATTGC and 5'-TCCAGCATCTTGTTCTGCTG with XmnI; rs2638526: 5'-TCACCTAATGGTGGGGAGAG and 5'-CTCACATTCACTGCCACCTG with HpyCH4IV; rs2070876: 5'-AGAACCACGAAGAGGCAAGC and 5'-AGAATGCTCTTCATCAGAGC with AciI [5].

## Results

### A microsatellite between *KRT8 *and *KRT18 *shows association with CF disease manifestation

**Figure 1:**
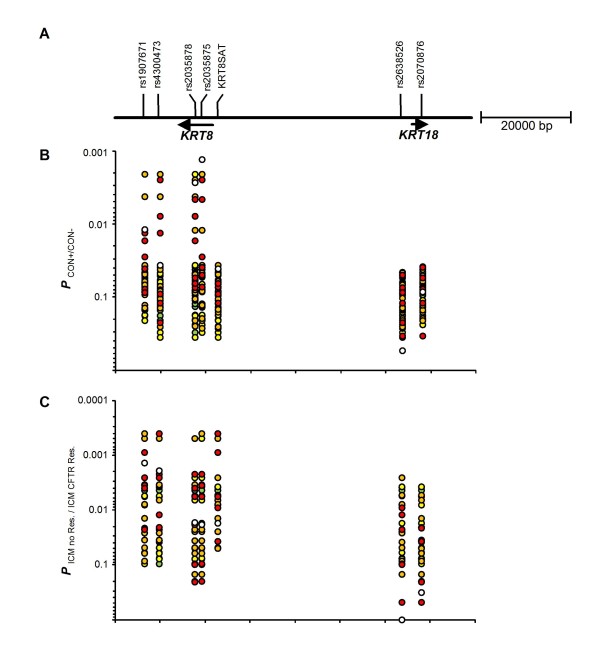
**Association study**. A: Map of the *KRT8*/*KRT18 *region on 12q13 showing the investigated microsatellite KRT8Sat and six SNPs. B, C: Results of the association study. Uncorrected P-values are shown for single markers (open circles), two- (red), three- (orange), four- (yellow) and five- (green) marker-haplotypes, describing adjacent and distant combinations of 6 SNPs and one microsatellite. Please note that some haplotype combinations are not visible in this plot as identical values will not show in an overlay. B: P values for comparison of haplotype distributions from concordant mildly affected (CON+; 13 families) to concordant severely affected (CON-; 12 families). P_best _= 0.00131 is observed for marker rs2035875. P_corr _= 0.0185 (corrected for simultaneous analysis of seven markers) [25]. C: P values for comparison of diplotype distributions from patients classified by their basic defect through intestinal current measurement (ICM). Comparison was done between patients who do not exhibit residual chloride secretion (ICM no Res., 14 families) and patients who show CFTR mediated residual chloride secretion (ICM CFTR Res., 22 families). P_best _= 0.0004 is observed for the two- marker-combination rs4300473-KRT8Sat and the three-marker-combinations rs4300473-rs2035875-KRT8Sat, rs1907671-rs2035875-KRT8Sat, rs1907671-rs4300473-KRT8Sat as well as the four-marker-combination rs1907671-rs4300473-rs2035875-KRT8Sat. P_corr _= 0.0069 (corrected for simultaneous analysis of seven markers) [25].

We have genotyped KRT8Sat, a dinucleotide repeat located between *KRT8 *and *KRT18 *(Figure 1A). Allele distributions were significantly different comparing mildly (CON+) and severely (CON-) affected patient pairs (P_raw _= 0.0409) and genotype distributions were significantly different comparing patients without chloride secretion (ICM-no Res) in excised intestinal biopsies to F508del-*CFTR *homozygotes who exhibit CFTR-mediated residual chloride secretion (ICM-CFTR Res.) determined by intestinal current measurement (P_raw _= 0.0177).

To determine whether the association signal observed at KRT8Sat is due to variations in *KRT8 *and/or *KRT18*, we have next selected informative single nucleotide polymorphisms in these equally plausible candidate genes [19].

### Intragenic SNPs in *KRT8 *allocate the association signal to CF basic defect and disease severity

Four SNPs in and near *KRT8 *and two SNPs in and near *KRT18 *have been evaluated for association with haplotype (phenotypic contrast CON-/CON+) and diplotype (phenotypic contrast ICM-no Res./ICM-CFTR Res.) distributions (Figure 1B,C). In both analyses, *KRT8 *markers provide smaller P values than *KRT18 *markers. Single marker analyses of rs2638526 and rs2070876 as well as the resulting two-marker combination for these variants within and near *KRT18 *did not reach significance. Those haplotype or diplotype distributions including one or both of these *KRT18 *markers which exceed the threshold of a 5% error probability are higher-order marker combinations that include at least KRT8Sat and/or one of the SNPs intragenic to *KRT8*, spanning the entire intragenic region and are inconclusive for mapping purposes (Figure 1B,C). In contrast, many single marker analyses and marker combinations restricted to the four SNPs rs1907671, rs4300473, rs2035878 and rs2035875 which are localized within or near *KRT8 *reach significance (Figure 1B,C). Within *KRT8*, linkage disequilibrium is strong with D' between any two of the four SNPs ranging from 0.901 to 1.000 (mean 0.962). SNP pairs composed of any of the four *KRT8 *SBNPs and any of the two *KRT18 *SNPs show D' values from 0.370 to 0.487 (mean 0.435). Thus, the two *KRT *genes reside on different haplotype blocks of the genome. We conclude that the causative variant responsible for the allelic association with CF disease severity and the CF basic defect which we observed at KRT8Sat is likely to be found in *KRT8 *and not in *KRT18*. Both association signals remain significant after correction for multiple testing with 7 markers (P_corr _= 0.0185 for contrast CON-/CON+ and P_corr _= 0.0069 for contrast ICM-no Res./ICM-CFTR Res.).

### The major contrasting *KRT8 *haplotypes constitute a dominant and a recessive allele which determine the manifestation of the CF basic defect

We have calculated haplotype frequencies in the entire patient population of 171 F508del-CFTR homozygotes from 101 families (Table 1). 90% of *KRT8 *chromosomes are accounted for by the two rs1907671-rs4300473-rs2035878-rs2035875 haplotypes 1122 and 2211 which occur on 43% and 48% of transmitted chromosomes (Table 1). Next, we have reviewed the haplotype and diplotype distributions among or patient subsamples to assign the risk and the benign allele (contrast CON-/CON+) and to deduce the mode of inheritance for the manifestation of CFTR mediated residual chloride secretion (contrast ICM-no Res./ICM-CFTR Res.). As the frequency of allele 2211 is elevated among the mildly affected patient pairs CON+ in comparison to the CON- sample, we conclude that 2211 is the benign and 1122 the severe allele at KRT8 (Table 1). The presumed mild allele 2211 is also observed more frequently among patients with CFTR mediated residual current while the frequency of the putative risk allele 1122 is elevated among patients who do not exhibit any chloride secretion (Table 1). In support of these conclusions, a deviation from the expectancy frequencies derived under the condition of the Hardy Weinberg-Law are observed among mildly affected patient pairs who are more frequently homozygous for the haplotype 2211 and among patients who do not exhibit any chloride secretion who are more frequently homozygous for the allele 1122 (Table 2). Moreover, 57% of patients who do not display residual secretion are homozygotes for the *KRT8 *haplotype 1122 while 77% are heterozygous or homozygous carriers for the putative mild *KRT8 *haplotype 2211 (Table 2). We deduce from this observation that out of the two frequent contrasting KRT8 haplotypes, 1122 is a recessive allele and 2211 is a dominant allele. In other words, carriership of one benign *KRT8 *haplotype 2211 allele is sufficient for the modification of the CF basic defect in F508del-*CFTR *homozygotes.

**Table 1:** *KRT8 *haplotype distributions

*KRT8 *haplotype rs1907671-rs4300473-rs2035878-rs2035875	T	CON-	CON+	**ICM no Res**.	**ICM CFTR Res**.
1122	0.428	0.515	0.287	0.731	0.394
2211	0.473	0.399	0.670	0.269	0.526
other pooled	0.099	0.086	0.043	< 0.001	0.080

*P*		P_raw _= 0.0028; P_corr _= 0.0051		P_raw _= 0.0151; P_corr _= 0.0049	

Alleles at all PCR-RFLP typed SNPs are named for absence (allele 1) or presence (allele 2) of diagnostic restriction site [5]; T: transmitted chromosomes of the entire patient population of 101 CF families (171 F508del-*CFTR *homozygotes) [5]; CON+: concordant mildly affected patient pair (13 families) [5]; CON-: concordant severely affected patient pair (12 families) [5]; ICM Res.: patients who exhibit CFTR-mediated residual chloride secretion in ICM (intestinal current measurement; 22 families) [5]; ICM no Res.: patients who do not exhibit residual chloride secretion in ICM (intestinal current measurement; 14 families) [5]; Praw: observed uncorrected P-value; Pcorr: corrected for multiple testing of 4 SNPs [25]

**Table 2:** *KRT8 *diplotype distributions

*KRT8 *diplotype rs1907671-rs4300473-rs2035878-rs2035875	E	CON-	CON+	**ICM no Res**.	**ICM CFTR Res**.
1122/1122	0.183	0.141	0.047	0.570	0.045
1122/2211	0.405	0.588	0.439	0.357	0.589
2211/2211	0.224	0.101	0.428	0.071	0.182
other pooled	0.188	0.170	0.086	0.002	0.184

*P*		P_raw _= 0.0319; P_corr _= 0.0252		P_raw _= 0.0049; P_corr _= 0.0035	

Alleles at all PCR-RFLP typed SNPs are named for absence (allele 1) or presence (allele 2) of diagnostic restriction site; E: expectancy values, based on Hardy-Weinberg-Law, relying on haplotype frequencies given in Table 1 for entire cohort (101 CF families); CON+: concordant mildly affected patient pair (13 families) [5]; CON-: concordant severely affected patient pair (12 families) [5]; ICM Res.: patients who exhibit CFTR-mediated residual chloride secretion in ICM (intestinal current measurement; 22 families) [5]; ICM no Res.: patients who do not exhibit residual chloride secretion in ICM (intestinal current measurement; 14 families) [5]; P_raw_: observed uncorrected P-value; P_corr_: corrected for multiple testing of 4 SNPs [25]

### The mild *KRT8 *haplotype is phylogenetically old

**Figure 2:**
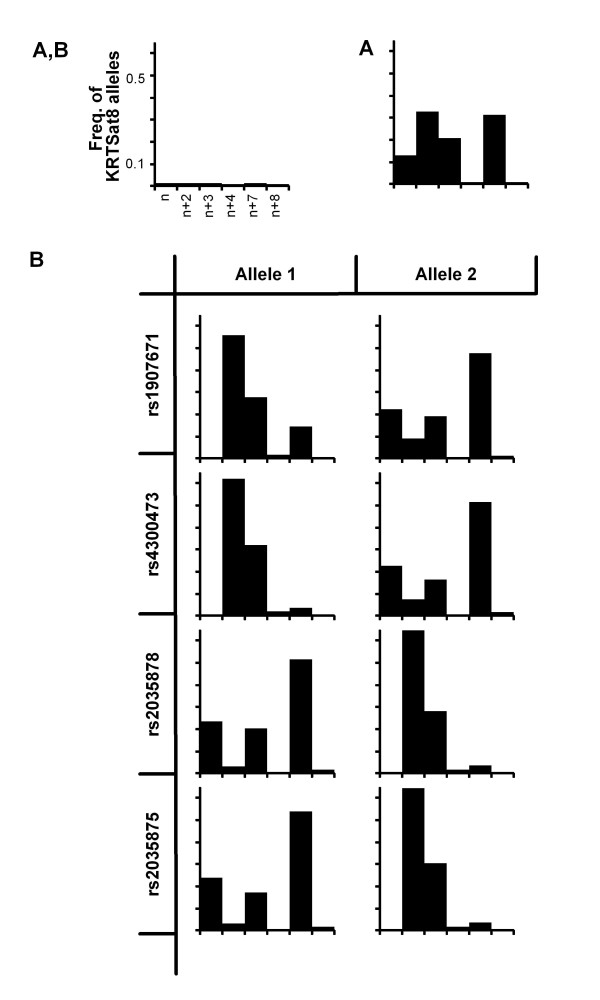
**Mikrosatellite KRT8Sat allele distributions on *KRT8 *SNP background**. Pictograms show the allele distribution of KRT8Sat. The reference sequence for the dinucleotide repeat KRT8Sat is (CA)_12_(TA)(CA)_2_(TA)(CA)_4 _starting at position 15442813 on contig NT_029419. Alleles were calibrated in arbitrary repeat units using an invariant set of controls for all analyses. Six alleles were observed in the entire population, differing in size by one, three, four, seven and eight dinucleotide units in reference to the smallest allele observed. A: Allele distribution at KRT8Sat, observed among 101 CF families with a total of 171 patients. B: Distribution of KRT8Sat alleles on SNP allele background, given for allele 1 at SNP markers (left column) and allele 2 at SNP markers (right column). Alleles at all PCR-RFLP typed SNPs are named for absence (allele 1) or presence (allele 2) of diagnostic restriction site. SNPs rs1907671 (top row), rs4300473 (2^nd ^row), rs2035878 (3^rd ^row) and rs2035875 (bottom row) are shown. See text for details.

We wanted to know which of the two frequent KRT8 haplotypes is older and have analyzed the four two-marker-haplotypes each composed of one out of the four *KRT8 *SNPs rs1907671, rs4300473, rs2035878 and rs2035875 in combination with the microsatellite KRT8Sat (Figure 2). Four major alleles, each present on more than 10% of chromosomes and all of them accounting for 98% of chromosomes are observed at the (CA)_n_-repeat KRT8Sat in the entire patient sample (Figure 2). SNP alleles 2 at rs1907671, 2 at rs4300473, 1 at rs2035878 and 1 at rs2035875 were observed to occur with three major microsatellite alleles that differ by more than one repeat motif in size, showing a broad distribution that has likely been generated by more than one recombination event (Figure 2). In contrast, the opposite SNP alleles 1 at rs1907671, 1 at rs4300473, 2 at rs2035878 and 2 at rs2035875 are observed together with two microsatellite alleles that differ by one repeat motif in size, one presumably having emerged from the other by an indel event in the microsatellite's primary sequence (Figure 2). Under the assumption that the KRT8Sat alleles themselves are functionally equivalent and neutral towards the studied phenotypes, we conclude that the benign *KRT8 *haplotype 2211 is older than the severe 1122 allele as one slippage event, explaining the KRT8Sat allele distribution on the 1122 background, requires fewer generations than the multiple recombination events that have generated the microsatellite's sequence pattern on 2211 alleles.

## Discussion

By genotyping F508del-*CFTR *homozygous sibpairs and unrelated index cases selected for their extreme clinical or electrophysiological phenotype, we have mapped a modifier for disease severity and CFTR mediated residual chloride secretion to *KRT8 *based on an initial interrogation with one informative microsatellite and a low-density-scan with six SNPs located within or near the two neighbouring and equally plausible candidate genes *KRT8 *and *KRT18 *(Figure 1, Tables 1, 2). As strong LD was observed at *KRT8*, we could assign the risk and the benign allele at this locus to the two similarly frequent major four-marker-haplotypes that account for about 90% of alleles in our patient population (Table 1). We have observed that the CF basic defect is determined via *KRT8 *whereby the phylogenetically older *KRT8 *haplotype 2211 is dominant for the manifestation of CFTR mediated residual chloride secretion and also recognized consistently as the benign modifier allele that is associated with a milder clinical phenotype among concordant CF sibpairs (Table 2, Figure 2). Accordingly, the phylogenetically younger contrasting haplotype 1122 constitutes the recessive risk allele (Table 2, Figure 2). As the phylogenetically older and dominant allele conveys CFTR mediated chloride secretion, indicative of proper targeting of CFTR to the epithelial apical membrane, we conclude that this condition has provided a selective advantage to human beings. Consistently, the younger recessive risk allele manifests only in the homozygous state.

The strategy employed here, i.e. the initial interrogation of a candidate gene and the subsequent fine-mapping using informative SNPs has been successfully applied before to identify CF modifying genes on 12p13 [26] and 19q13 [27]. In these two cases, haplotype-guided hierarchical fine-mapping [27] has allowed the identification of all possible causative variants on small genomic fragments of 2 kb, 3 kb and 7.5 kb [26,27]. For *KRT8*, we did not pursue our approach beyond the present stage to describe the difference between the two contrasting haplotypes comprehensively as the fragment between rs1907671 and rs2035875 already covers 13 kb due to the high LD observed and is likely to extend further at both sides, involving several kb of intergenic sequence with unknown function which will be difficult to annotate without further information on the molecular mechanism that determines its role as a CF modifier.

The type I cytokeratin KRT18 and the type II cytokeratin KRT8 are coexpressed and copolymerize to form intermediate filaments in single-layered epithelial cells. Both, *KRT8 *and *KRT18 *have been implicated before as candidate genes that modify the phenotype of the mutant F508del-CFTR, known to be defective in protein folding, processing and trafficking [10] by functional data: Davezac et al [19] have analyzed cell lines transfected with wt-CFTR and F508del-CFTR by differential two-dimensional electrophoresis and identified KRT8 and KRT18 to be differentially expressed.

It is conceivable that the role of the two opposing *KRT8 *alleles which have been identified as a benign and as a risk allele in this work are effective in the context of epithelial cell differentiation. Based on a mouse model that expresses human KRT8 on top of its murine homologue and curiously shares several pathologic characteristics with a transgenic mouse that expresses a dominant-negative mutant TGF-beta type II receptor, it has been suggested that the regulation of keratin expression could be related to neoplastic and/or inflammatory disorders [28]. As the mild KRT8 allele is associated with CFTR mediated residual chloride secretion among F508del-*CFTR *homozygotes, the KRT8/KRT18 heterodimeric intermediary filaments of the cytoskeleton may be an essential component for the proper targeting of CFTR to the apical membrane in epithelial cells.

## Conclusions

In conclusion, we have identified two frequent contrasting *KRT8 *haplotypes whereby these influence the manifestation of the CF basic defect as a dominant and as a recessive allele. The importance of these two *KRT8 *variants is substantiated by their association with CF disease severity. Our results on *KRT8 *exemplify that modifiers of CF disease severity can be recognized through their association with the CFTR-mediated basic defect, which is less likely to be prone to perturbations by environmental factors in comparison to lung function measurements which are frequently relied on as the sole outcome parameter in CF modifier analysis.

## Competing interests

The authors declare that they have no competing interests.

## Authors' contributions

Genotyping data, including the responsibility for the accuracy of primary data and its quality control, was generated by SH. Analysis and interpretation of genetic data was done by FS. TB is a consultant to the European CF twin and sibling study for statistics and genetic epidemiology. The concept of the study was designed by BT. All authors have revised the manuscript critically for content and have approved the final version.

## Pre-publication history

The pre-publication history for this paper can be accessed here:

http://www.biomedcentral.com/1471-2350/12/62/prepub
